# The triple burden: achieving sexual and reproductive health equity for young women with albinism in Rwanda

**DOI:** 10.11604/pamj.2025.52.167.50448

**Published:** 2025-12-17

**Authors:** Samuel Niyomuremyi, Jean Claude Sabato, Tayechalem Moges, Oluwaseun Ayoola Ojomo

**Affiliations:** 1 University of Global Health Equity, Kigali, Rwanda

**Keywords:** Albinism, sexual and reproductive health, intersectionality, stigma, disability, gender, Rwanda

## Abstract

Young women with albinism in Rwanda face a triple burden of intersecting stigma related to gender, disability, and albinism-specific myths, yet their sexual and reproductive health (SRH) needs remain largely invisible in research, policy, and practice. Despite Rwanda´s progress toward universal health coverage and disability inclusion, no published studies specifically examine SRH access, outcomes, or barriers among this population. Drawing on intersectionality theory and evidence from separate bodies of literature on gender, disability, and albinism in East Africa, this perspective article argues that overlapping discrimination likely creates unique and compounded obstacles to contraception, antenatal care, sexually transmitted infection (STI) services, and freedom from sexual violence. Cultural beliefs portraying albinism as a curse or conferring supernatural properties perpetuate social exclusion, internalized shame, and heightened vulnerability to gender-based violence. Visual impairment associated with albinism compounds accessibility challenges, while the absence of low-vision materials, genetic counselling, sun-protection guidance, and provider training on albinism further limits equitable care. National SRH and disability frameworks mention persons with disabilities broadly but lack tailored provisions for albinism. To achieve true health equity, Rwanda must make young women with albinism visible through disaggregated data collection, explicit integration into SRH policies, accessible materials and facilities, mandatory provider training, and community-led myth-busting initiatives. Primary research co-designed with women with albinism is urgently needed to document their lived experiences and evaluate interventions. Addressing these intersecting barriers will strengthen inclusive SRH services for all marginalized groups.

## Perspectives

Rwanda has made significant strides in public health advancements by establishing universal health coverage [1]. However, it remains unclear whether people with albinism have equitably benefited from these enhancements, as research specifically examining health coverage and service access for this population is lacking [2,3]. Albinism is a genetic condition characterized by reduced melanin production, resulting in pale skin, light-colored hair, and visual impairments [4]. It affects an estimated 1 in 1000 to 1 in 15,000 individuals in sub-Saharan Africa [5]. In Rwanda, the most recent data (2022 census) identifies 1,864 people with albinism, equivalent to approximately 1 in 7,106 of the Rwandan population [6]. Young women with albinism may face a triple burden, including gender-based violence and discrimination, disability-related stigma, exclusion from leadership roles, forced displacements based on harmful beliefs, and the unique vulnerabilities associated with albinism [7]. When these factors converge in the context of sexual and reproductive health, they can create a unique barrier, which is overlooked by both research and practice. This perspective article argues why the sexual and reproductive health (SRH) needs of young women with albinism demand immediate attention. Sexual and reproductive health refers to “a state of complete physical, mental, and social well-being in all matters relating to the reproductive system,” encompassing access to contraception, family planning, maternal and newborn health, prevention and treatment of sexually transmitted infections, and freedom from sexual violence and coercion [8]. In this article, we examine the intersectional barriers these young women likely face, highlight critical gaps in research and policy, and propose an agenda for action that centers their voices and experiences.

**The intersectionality of invisible barriers:** many people with disability experience disproportionately poor health outcomes and encounter significant barriers when seeking health services. Several of them are likely to die up to 20 years earlier, face more than twice the risk of developing conditions such as diabetes, stroke, or depression, and often struggle with inaccessible health facilities, barriers that can be up to six times more hindering [9]. The experiences of young women with albinism in accessing SRH care are likely shaped by intersectionality, a framework that recognizes how overlapping social identities create unique experiences of discrimination [10]. In this context, albinism-related stigma interacts with gender norms and disability-based prejudice to produce profound health inequities. Globally, an estimated 218 million women of reproductive age have an unmet need for modern contraception [11]. Additionally, 1 in 3 women experiences gender-based violence in their lifetime [12]. Young women face significant barriers to accessing SRH services, including fear of judgment, lack of confidentiality, provider bias, and cultural norms that impose shame and silence around female sexuality [13]. In many contexts, including Rwanda, these norms are particularly restrictive for young women and girls with disabilities, who are often seen as asexual or hypersexual, further deterring them from seeking contraception, STI testing, or antenatal care [14,15].

People with disabilities consistently report discrimination in healthcare settings, with providers often lacking training to address their specific needs [16]. Visual impairments associated with albinism require reasonable accommodations (e.g., large-print materials, verbal explanations) that are rarely provided in SRH settings. Beyond general disability discrimination, albinism carries unique cultural meanings. In Rwanda and across East Africa, myths persist that albinism is a curse, that people with albinism are not fully human, or that sexual intercourse with a person with albinism can cure HIV [17]. These beliefs fuel violence, social exclusion, and profound barriers to agency in sexual decision-making. While research has not specifically examined the combined effects of gender, disability, and albinism on SRH access, evidence from three separate bodies of literature suggests that women who embody gender, disability, and albinism-specific discrimination may face uniquely compounded barriers that are qualitatively different from and more severe than those faced by women without disabilities or men with albinism [18,19-21]. Studies on gender show that young women experience judgment, limited autonomy, and provider bias in accessing SRH services [13,18,20,21]. Research on disability demonstrates that women with disabilities encounter research on other forms of discrimination, inaccessible information, and inadequate provider training [16]. Additionally, albinism-focused studies from East Africa document harmful myths, social exclusion, and heightened vulnerability to sexual violence [17,22,23]. Together, these findings imply that women who embody all three identities may experience layered barriers not captured in single-identity research.

**Silence in sexual and reproductive health:** to our knowledge, an extensive literature search has revealed no published study that specifically examined SRH access among young women with albinism in Rwanda. This absence may reflect the compounding invisibility of this population. They are overlooked in disability research (which rarely disaggregates by gender or specific conditions), in women's health research (which seldom includes disability perspectives), and in albinism research (which has primarily focused on dermatological and ophthalmological concerns rather than reproductive health). The available evidence is limited, primarily based on a few key findings. Research in Tanzania and Malawi documents myths about albinism bringing wealth or healing but does not explore access to SRH services [24]. Studies on women's SRH barriers identify stigma and provider bias as major deterrents but do not include the experiences of women with albinism [16].

**The Rwandan context: persistent gaps:** despite Rwanda´s National Policy for Persons with Disabilities (2018) and its commitment to inclusion, people with albinism remain largely invisible in implementation guidance. There are no tailored measures for their visual, dermatological, and genetic needs evident in disability and health programming [25]. National sexual and reproductive health (SRH) frameworks, such as the Family Planning and Adolescent Sexual and Reproductive Health Strategic Plan 2018-2024, treat albinism only under the general umbrella of persons with disabilities. They contain no dedicated provisions for low-vision-accessible materials, sun-protection counselling in prenatal or family-planning services, genetic counselling on autosomal recessive inheritance, or protocols to manage elevated skin-cancer risk during pregnancy [26]. Cultural myths portraying albinism as a curse, a burden, or a sign of misfortune persist in parts of Rwanda, fuelling social isolation, and research on stigma suggests these attitudes may contribute to internalized shame and fear of judgement or verbal abuse in health facilities. While direct research on health-seeking behavior among women with albinism is lacking, these attitudes may deter women and girls with albinism from seeking contraception, antenatal care, or reproductive counselling, potentially undermining their reproductive autonomy [27]. In the absence of a specific national report on albinism and SRH in Rwanda, this raises the broader question of whether the exclusion of people with disabilities from SRH decision-making and education means that individuals with albinism are similarly overlooked or even more neglected as a result of intersecting vulnerabilities. The fact that disability inclusion is insufficiently implemented in SRH services signals that women with albinism are doubly disadvantaged by disability invisibility and by albinism-specific stigma.

**Why this matters now: a call to action:** countries are just 40 steps away from achieving health equity for people with disabilities. States have a legal duty to eliminate existing health disparities and ensure that people with disabilities can fully exercise their inherent right to the highest attainable standard of health. Tackling these inequalities strengthens health systems for everyone by promoting universality, people-centeredness, and non-discrimination in health services, ultimately making services and public health initiatives more effective and responsive to the needs of all. In addition, investing in disability-inclusive prevention and non-communicable disease care delivers substantial benefits, generating an estimated US$10 return for every US$1 invested [9]. The first step toward equity is making the invisible visible. Young women with albinism in Rwanda deserve SRH services free from the shadows of stigma and discrimination. Below, we outline key recommendations that can guide Rwanda´s efforts to build an inclusive SRH system.

### Recommendations

**Policymakers:** i) integrate albinism explicitly into national SRH policies, guidelines, and National Reproductive, Maternal, Newborn, Child and Adolescent Health (RMNCAH) policy strategies; ii) mandate the collection of disability-disaggregated data, including albinism, within health information systems; iii) fund the development and distribution of accessible SRH materials (large-print, audio, tactile); iv) expand social protection schemes to subsidize high-factor sunscreen and protective clothing for women with albinism attending SRH services; v) integrate albinism into national SRH policies and guidelines, including RMNCAH strategies and disability-inclusion frameworks.

**Healthcare providers and health facilities:** i) deliver mandatory training on albinism (vision-related needs, sun-exposure risks in pregnancy, respectful communication, debunking myths); ii) adapt facilities and materials to be low-vision-friendly (signage, forms, educational tools); iii) strengthen confidentiality protocols and integrate psychosocial support for stigma-related trauma or sexual violence.

**Community leaders and civil society:** i) launch targeted myth-busting campaigns using radio, community theater, and schools; ii) elevate positive role models-women with albinism who speak openly about SRH and rights; iii) strengthen protection networks, especially in rural areas, in collaboration with organizations such as the Rwanda Albinism Network.

**Researchers and academic institutions:** i) conduct primary research to document SRH outcomes, access barriers, and gendered experiences among young women with albinism, as a critical first step given the complete absence of evidence in this area. ii) evaluate interventions that aim to improve accessibility, provider training, or myth reduction; iii) include women with albinism as co-researchers or community advisors, ensuring the research agenda reflects their lived realities.
